# Micro-spectroscopy on silicon wafers and solar cells

**DOI:** 10.1186/1556-276X-6-197

**Published:** 2011-03-04

**Authors:** Paul Gundel, Martin C Schubert, Friedemann D Heinz, Robert Woehl, Jan Benick, Johannes A Giesecke, Dominik Suwito, Wilhelm Warta

**Affiliations:** 1 Fraunhofer Institute for Solar Energy Systems (ISE), Heidenhofstr. 2, 79110 Freiburg, Germany

## Abstract

Micro-Raman (μRS) and micro-photoluminescence spectroscopy (μPLS) are demonstrated as valuable characterization techniques for fundamental research on silicon as well as for technological issues in the photovoltaic production. We measure the quantitative carrier recombination lifetime and the doping density with submicron resolution by μPLS and μRS. μPLS utilizes the carrier diffusion from a point excitation source and μRS the hole density-dependent Fano resonances of the first order Raman peak. This is demonstrated on micro defects in multicrystalline silicon. In comparison with the stress measurement by μRS, these measurements reveal the influence of stress on the recombination activity of metal precipitates. This can be attributed to the strong stress dependence of the carrier mobility (piezoresistance) of silicon. With the aim of evaluating technological process steps, Fano resonances in μRS measurements are analyzed for the determination of the doping density and the carrier lifetime in selective emitters, laser fired doping structures, and back surface fields, while μPLS can show the micron-sized damage induced by the respective processes.

## Introduction

Silicon solar cells contribute by far the largest share to the world's photovoltaic facilities [1]. An important property to classify these silicon solar cells is the base material, where two fundamentally different approaches can be observed in the photovoltaic industry: multicrystalline and monocrystalline cells. While the fabrication of monocrystalline silicon is more expensive, the efficiency potential of these cells is higher. The world record efficiency for monocrystalline silicon solar cells is 25.0% [2] and 20.4% for multicrystalline silicon [3]. As different as these base materials are as different as the arising challenges in the industrial production: To realize the efficiency potential and to lower the price per Watt-peak of monocrystalline cells, sophisticated cell structures with doping microstructures including selective emitters, laser fired back surface fields [4], and backside contacts have been introduced and are partly already adopted in the industrial production. For multicrystalline silicon, the photovoltaic industry tries either to use less pure and cheaper silicon ("upgraded metallurgical grade silicon") and to improve this material during the solar cell process by high temperature and gettering steps, to reduce the costs, or to use multicrystalline material with low defect densities to increase the efficiency potential. From these strategies, two important fields of microscopic research emerge: the detailed characterization and improvement of doping microstructures and the research on microdefects, which limit the performance of multicrystalline cells. Both fields require the development and application of electrical characterization techniques which provide a high spatial resolution of at least 1 μm.

In this paper, we demonstrate the latest advances on these research fields, which are based on micro-Raman spectroscopy (μRS) and micro-photoluminescence spectroscopy (μPLS). First, we will introduce the measurement techniques and how the important parameters doping density, carrier lifetime and mechanical stress can be extracted from both techniques with a spatial resolution of down to 500 nm. In the second part, we will apply these techniques (1) for the characterization of technological doping microstructures and (2) for the fundamental research on the recombination activity of precipitates.

## Experimental setup and samples

μRS and μPLS are based on the same scanning confocal microscope, which features a 532 nm laser as point excitation source, a ×50 lens with a numerical aperture of 0.65 for μPLS and a ×100 lens with a numerical aperture of 0.9 for highly resolved μRS measurements. The spotsize of the laser is less than 500 nm in diameter and the power on the sample can be varied between 0 and 27 mW. Details on the setup can be found in [5,6].

The sample surfaces for the multicrystalline samples and the cross-sections of the back surface field (BSF) and the laser-processed BSF were polished mechanically. No surface passivation has been applied to all samples. The multicrystalline wafer is 1.5 × 10^16 ^cm^-3 ^boron doped and was intentionally contaminated with nickel.

## Quantitative Raman and photoluminescence spectroscopy

In this section, the techniques to quantitatively determine the doping density, the Shockley-Read-Hall lifetime, and the residual stress with micron resolution are presented in the two following subsections. The Shockley-Read-Hall lifetime is highly correlated to the efficiency of multicrystalline silicon solar cells.

### Micro-photoluminescence spectroscopy

**Figure 1:**
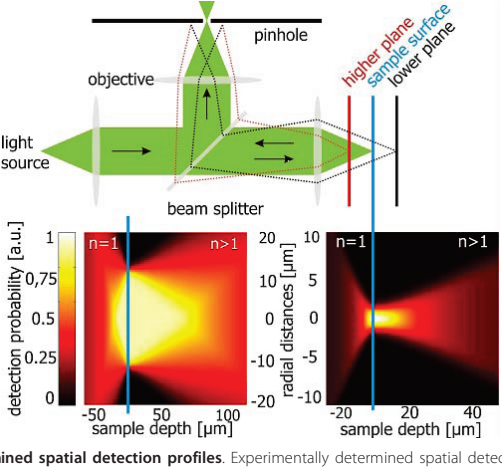
**Experimentally determined spatial detection profiles**. Experimentally determined spatial detection profiles with the big and the small pinhole corrected for the refractive index outside and inside of the sample. The *n *values refer to the refractive index in silicon and air.

The requirement for the high resolution of about 1 μm of all of the discussed techniques is to measure under high injection conditions, since the carrier diffusion length has to be in the order of the spatial resolution or lower. This is typically the case only under high injection conditions, where Auger recombination limits the diffusion length to 1 μm or less. The physical principle behind the quantitative determination of doping density and Shockley-Read-Hall lifetime by μPLS is to measure the depth profile of the injection density and to compare the measurement with simulations. The depth profile is measured by varying the pinhole size of the confocal microscope, which allows to measure with different spatial detection profiles. We execute two measurements with a pinhole size of 100 and 1,000 μm, respectively. The detection profiles of both pinhole sizes are experimentally determined by scanning a pre-breakdown site with a diameter of less than 550 nm (Figure 1).

By dividing the PL intensity around the center of the band-to-band PL peak *I_1 _*(large pinhole) by the PL intensity *I_2 _*(small pinhole), we obtain information about the depth profile. Using the ratio of two measurements has the advantage that unknown parameters such as the absolute quantum efficiency of the detector system and the emissivity of the sample surface cancel out. The measured ratio *Q = I_1_/I_2 _*is compared to numerical two dimensional simulations of the injection density and the resulting *Q*. We call these techniques micro-photoluminescence lifetime mapping [7] and micro-doping density mapping [8].

**Figure 2:**
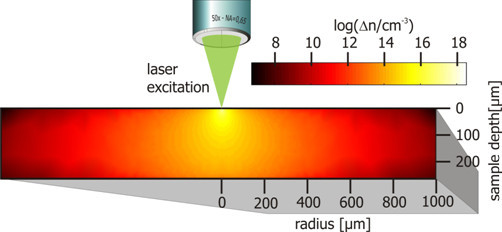
**Example for the simulated injection density**. The injection density drops sharply within a few microns from the point of excitations.

**Figure 3:**
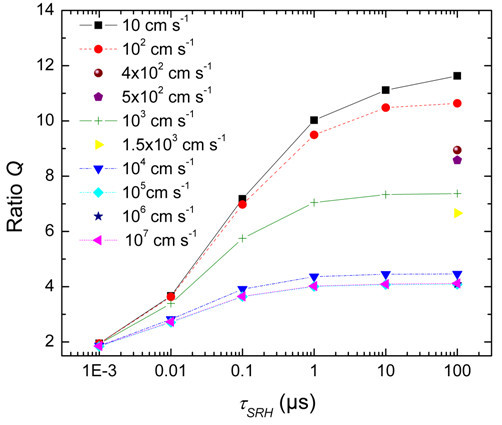
**Graphic representation of calibration table**. Graphic representation of a part of the calibration table for a 1.5 × 10^16^-cm^-3 ^p-doped sample with different surface recombination velocities. From the ratio *Q*, which is monotonically increasing with the Shockley-Read-Hall lifetime *τ*_SRH_, we can directly determine *τ*_SRH_. In analogy to this calibration table, a table for the determination of the doping density can be plotted

By this comparison, the Shockley-Read-Hall lifetime and the doping density can be extracted. An example for the simulated injection density in the sample and a graphic representation of a part of the calibration table for the lifetime are shown in Figures 2 and 3.

Furthermore, μPLS can be utilized to measure the bandgap energy. Since the bandgap energy depends on the residual stress [9,10] and the doping density, these parameters can be extracted from the μPLS measurement. For this the PL spectrum at 300 K is empirically fitted with three overlapping Gaussians with fixed relative spectral distances and the relative peak shift is extracted. From the relative peak shift, the stress level can be calculated if the doping density is homogeneous (variations below 10^17 ^cm^-3^, where the influence on the bandgap energy becomes significant). In [6], we could show that the measured stress is in agreement with μRS stress measurements. If no stress is present, the doping density can be estimated.

### Micro-Raman spectroscopy

**Figure 4:**
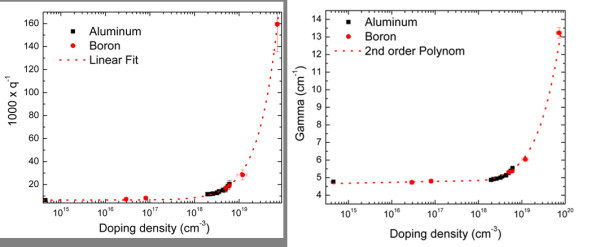
**Hole density dependence of *q *and *Γ***. **(a) **Hole density (doping density) against 1,000 × *q*^-1^·*q*^-1 ^is proportional to the hole density. The doping element (aluminum and boron) has no significant effect on the calibration. **(b) **Hole density (doping density) against line width *Γ*. The fit shows a quadratic dependence of *Γ *on the hole density.

The measurement of stress by μRS is well known and is not discussed here. In [11,12], excellent descriptions of this technique can be found. We are focusing here on the determination of the doping density and the Shockley-Read-Hall lifetime. Becker et al. [13] demonstrated the doping density measurement with μRS. This technique is based on the Fano resonance between the Raman active optical phonons and the free holes [14]. According to Fano, the shape of the first order Raman peak in wavenumbers Ω is:

(1) $$\text{I}\Omega =\frac{{\left[q+2(\Omega -\Omega_{\text{peak}})\Gamma^{-1}\right]}^{2}}{1+{\left[2(\Omega -\Omega_{\text{peak}})\Gamma^{-1}\right]}^{2}}$$

with the Fano asymmetry parameter *q *and the line width *Γ*. *Γ *and *q*^-1 ^increase monotonically with the hole density [15] and thus, can be used to measure the hole density. To calibrate both parameters with the hole density, we measured the Raman spectra of samples with known doping densities at 0.7 mW laser power on the sample and fitted the first order Raman peak with equation 1. From the fits, we can extract the hole density dependence of *q *and *Γ *(Figure 4).

In samples with unknown doping densities, the calibration curves are used to determine the doping density. Since *Γ *is more robust against fitting errors than *q*, we rely on this parameter for the measurements below.

At high injection, the Fano resonance is not solely governed by the doping density but also by the injected holes. With simulations in analogy to [7,8] and the calibration tables in Figure 4, the Shockley-Read-Hall lifetime can be measured at injection densities above 10^18 ^cm^-3^. An excellent agreement between μPLS and μRS Fano measurements was demonstrated in [16].

The advantage of μRS compared to μPLS is the higher spatial resolution of 500 nm or less. μPLS offers the advantages to measure not only p-type doping but also n-type doping and the measurements are typically less noisy. Furthermore μPLS has the ability to measure the defect luminescence within the same measurement.

## Aluminum back surface field

**Figure 5:**
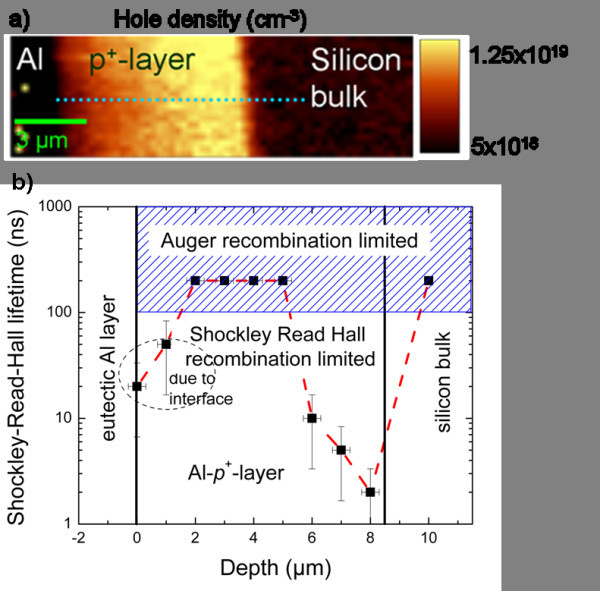
**Hole density and lifetime values in the BSF. **The Raman-Fano-measured hole density in the BSF (p+-layer) **(a) **and the resulting effective Shockley-Read-Hall lifetime at high injection** (b). **Lifetime values greater than 200 ns mean that the lifetime is solely limited by Auger recombination.

The SRH lifetime measurement along a line scan through the BSF (p^+^-layer) of a monocrystalline silicon solar cell is exemplified here. The doping density profile was measured with electrochemical capacitance voltage and is taken into account in the simulation for the lifetime determination. The lifetime within the BSF is crucial for the solar cell performance. An average value was determined to be 120 ns by Schmidt et al. [17]. For our spatially resolved measurements, we use μRS on a cross section of the BSF. The measured hole density in the BSF at a laser power of 27 mW is depicted in Figure 5a.

**Figure 6:**
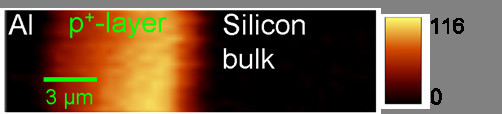
**Intensity of the defect luminescence at 1,250 nm in the BSF**. The intensity is clearly increased at the right side of the BSF, which indicates a higher defect density here. This could cause the low lifetimes at the interface between BSF and silicon bulk.

Figure 5b shows the effective Shockley-Read-Hall lifetime along a linescan. Lifetime values greater than 200 ns mean that the lifetime is solely limited by Auger recombination under the measurement conditions. At the interfaces between BSF and aluminum contact and BSF and silicon bulk we detected low SRH lifetimes. While this may be caused by the high surface recombination at the aluminum contact, the nature at the second interface is less clear. Therefore, we investigated this area with μPLS and showed an increased defect luminescence at 1,250 nm in this area (Figure 6), which is an indication for a higher defect density in this area, which could cause the drop in lifetime. Defect luminescence at 1,250 nm was observed in previous experiments on multicrystalline silicon at recombination active defects [6]. A more detailed analysis of the BSF can be found in [18].

## Laser doping from a dopant containing passivation layer (PassDop)

**Figure 7:**
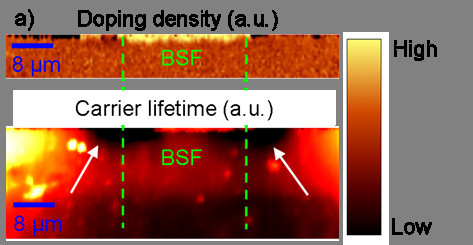
**Doping density and carrier lifetime in PassDop sample**. **(a) **Qualitative doping density, which is significantly increased in the laser-induced doping region (at the upper surface between the green lines) and **(b) **damage (map of the μPLS intensity) at the edges of the laser affected region which decreases the lifetime (white arrows).

In this section, we qualitatively analyze the cross section of a laser-induced BSF. Local highly doped regions are prepared by point wise laser irradiation of a silicon surface which was previously coated by a phosphorous containing passivation layer [4]. By laser irradiation, the dopant source and the underlying silicon is molten and a phosphorous diffusion in the liquid volume takes place resulting in a local, highly n-doped region. This high doping density underneath the subsequently evaporated metal contacts effectively suppresses recombination at the contact points and furthermore results in a low contact resistance. The high doping is visible by a shift of the PL peak to higher wavelengths (Figure 7a). The PL shift is caused by the decrease of the bandgap at higher doping densities. In Figure 7b, the micron-sized damage, which is caused by the laser process, can be seen at the edges of the laser processed area (white arrows) by a qualitative μPLS image map. This damage at the edges could be caused by the strong thermal gradient in this region during the laser firing. Another reason for the visibility of the damage is that there is no back surface field at the edges, which could passivate the damaged region. This shows the special care which has to be taken for the process laser profile in order to minimize the thermal stress in the edge regions.

## Multicrystalline silicon

**Figure 8:**
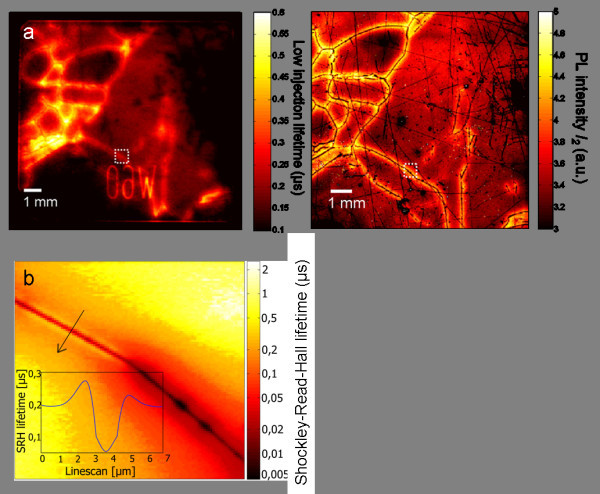
**Measurements on multicrytalline silicon**. **(a) **PL intensity *I_1 _*(left side) in comparison to a PL imaging measured lifetime (right side) of the same wafer. Both measurements are in good qualitative agreement. An excerpt in the white square is further analyzed in (b) In both images denuded zones of 100-μm width with higher lifetimes are visible around the dark grain boundaries. **(b) **Micro-photoluminescence lifetime map of the quantitative Shockley-Read-Hall lifetime. The map shows three grain boundaries with distinctively different recombination properties. The upper right grain boundary is almost recombination inactive and hardly visible, whereas the grain boundary on the lower right side is highly recombination active, which can be attributed to a strong metal precipitate decoration.

After demonstrating the applicability of μRS and μPLS on technological structures, we continue with measurements on defects in multicrystalline silicon. For this, a 1 × 1 cm^2 ^wafer is measured with micro-photoluminescence lifetime mapping. The PL intensity *I_1 _*with the large diameter is compared in Figure 8a to a PL imaging measurement. PL imaging is used here only for comparison and is explained in [19]. The images show a good qualitative agreement, even though μPLS measures under high injection and PL imaging measures in the low injection regime. This is due to the fact that high and low injection lifetimes are both proportional to the inverse defect density [20]. This highlights the usefulness of μPLS for the characterization of solar cells, which are typically working under low injection conditions. These results are discussed in detail in [7].

Figure 8b shows the Shockley-Read-Hall lifetime on a 100 × 100 μm^2 ^area at the triple point of three grain boundaries, which was measured by micro-photoluminescence lifetime mapping. The measurement shows the strongly different recombination activities of the three grain boundaries and reveals micron-sized denuded zones around the left grain boundary. The linescan across this grain boundary highlights the spatial resolution of micro-photoluminescence lifetime mapping. Micron-sized denuded zones could not be detected prior to the application of μPLS and μRS. The origin could be slowly diffusing impurities, which are internally gettered at the grain boundary during the block casting, which cleans the area around the grain boundary from these impurities. The lower right grain boundaries are highly recombination active, which is probably caused by a high metal decoration. Metal precipitates are also the most likely origin of the round structures along this grain boundary.

## Stress and recombination activity

The influence of stress on the recombination activity of metal precipitates is so far not known but often discussed. In this section, we will show experimental evidence that tensile stress increases and compressive stress reduces the recombination activity. For this, we map the areas around nickel precipitates, which are close to the wafer surface with μRS and extract the stress and the hole density. From the hole density, we calculate the hole density contrast *C*_RS _in analogy to the well-known EBIC contrast as measure for the recombination activity:

(2) $$C_{\text{RS}}=1-\frac{p}{p_{\max}},$$

with the maximum measured hole density *p*_max _and the hole density *p*.

**Figure 9:**
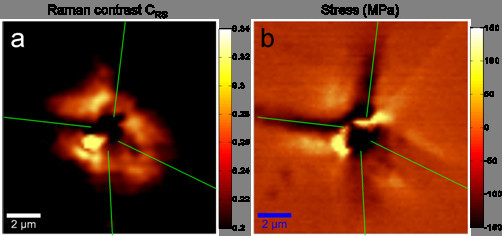
**Hole density contrast and stress around a nickel precipitate**. The green lines mark the directions of high compressive (negative) stress, which tend to show a lower hole density contrast (recombination activity). In areas of high tensile (positive) stress, the hole density contrast is increased (higher recombination activity).

Figure 9 shows, that high compressive stress correlates with lower recombination activities along the lines of high compressive stress and that high tensile stress correlates with higher recombination activities. This effect can be explained by the strong piezoresistance of silicon [21]: The carrier flux to the precipitate surface with its high surface recombination velocity [22,23] is proportional to the carrier mobility [24]. This change in mobility increases/reduces the carrier flux for tensile/compressive stress and hence, leads to a high/lower recombination activity in the respective directions. Another origin of the observed correlation between stress and recombination activity could be the formation of dislocations due to stress. However this formation would relax the stress and thus lead to a reduction of the correlation between stress and recombination activity. Details on the impact of stress on the recombination activity and a quantitative analysis can be found in [25,26].

## Conclusions

We presented an overview about the most recent developments of micro-Raman (μRS) and micro-photoluminescence spectroscopy (μPLS) and their successful application on technological microstructures and on fundamental problems of recombination at defects in silicon. We demonstrated the high resolution (< 1 μm) measurement of (1) the Shockley-Read-Hall lifetime by μRS and μPLS, (2) of the doping density by μRS and μPLS, and (3) of stress with both methods.

μRS has the advantage of a higher spatial resolution (about 0.5 μm compared to 0.8 μm) and is not influenced by defect luminescence, which can make the extraction of the bandgap energy and thus of the doping density and the stress from PL measurements difficult. μPLS has the advantages to be able to measure both n- and p-type doping and exhibits less noise in carrier lifetime measurements for comparable measurement times. Furthermore, the analysis of the defect luminescence can give a deeper insight in the carrier lifetime limiting defects.

We were able to detect high recombination activities within an aluminum-doped back surface field and the damage caused by a laser firing contact process, which shows ways to improve the processes.

On multicrystalline silicon, we investigated the recombination activity of grain boundaries and were able to measure micron-sized denuded zones around a grain boundary. We could explain the observed effect that recombination activity is significantly increased by tensile stress and reduced by compressive stress, by the high piezoresistivity of silicon.

## Competing interests

The authors declare that they have no competing interests.

## Authors' contributions

PG designed the study, carried out the μRS and μPLS measurements, participated in the simulations, and drafted the manuscript. MCS supervised the experiments and simulations. FDS participated in the simulations and carried out the lifetime measurement at the triple point. RW prepared the back surface field samples and assisted in the back surface field data interpretation. JB prepared the samples for the calibration of the Fano resonance. JAG performed the quantitative PL imaging measurement. DS prepared the PassDop samples and participated in the interpretation of the results on these samples. WW supervised the project work. All authors read and approved the final manuscript.
